# Editorial: Pyrophosphates and Polyphosphates in Plants and Microorganisms

**DOI:** 10.3389/fpls.2021.653416

**Published:** 2021-03-26

**Authors:** José R. Pérez-Castiñeira, Roberto Docampo, Tatsuhiro Ezawa, Aurelio Serrano

**Affiliations:** ^1^Instituto de Bioquímica Vegetal y Fotosíntesis, Universidad de Sevilla-CSIC, Sevilla, Spain; ^2^Department of Cellular Biology and Center for Tropical and Emerging Global Diseases, University of Georgia, Athens, GA, United States; ^3^Graduate School of Agriculture, Hokkaido University, Sapporo, Japan

**Keywords:** pyrophosphate, polyphosphates, phosphate metabolism, PPI, polyP

Phosphorus is the fifth most abundant chemical element in living cells. Microorganisms and plants take up phosphorus as dissolved (ortho)phosphate (Pi), that is often limited due to the formation of sparingly soluble complexes in soil; on the other hand, overapplication of phosphate fertilizer generally leads to the problems of eutrophication (diCenzo et al., 2017). Phosphorus usually occurs *in vivo* as free Pi or forming esters or diesters in metabolites and macromolecules. Protein phosphorylation also controls major metabolic pathways and cell division cycle (Li et al., 2016). Phosphate anion can react with another, releasing a molecule of water and producing a dimer, **pyrophosphate** (PPi, P_2_${\text{O}}_{7}^{4-}$). More Pi residues may be added to PPi by means of this linkage, known as a “phosphoanhydride bond,” thus producing **polyphosphate** (polyP). Hydrolysis of phosphoanhydride bonds is thermodynamically favorable and kinetically slow, consequently, PPi and polyP are used for energy transfer and storage in many organisms. PPi and polyP also participate in metabolites like nucleoside triphosphate, inositol pyrophosphate, or activated isoprene.

PPi is produced from ATP in many reactions, such as protein, RNA and DNA biosynthesis. Removal of PPi by hydrolysis allows the shifting of anabolic reactions toward biosynthesis, thus regulating biosynthetic fluxes and allowing recycling of Pi by incorporating it to ADP to form ATP. This links PPi metabolism with the control of the cellular energy status. PPi hydrolysis can be coupled to H^+^ or Na^+^-translocation across biological membranes by the membrane-bound H^+^-translocating and Na^+^-translocating inorganic pyrophosphatases (H^+^-PPases and Na^+^-PPases), respectively. H^+^-PPases occur in all members of the eukaryotic green evolutionary lineage (from unicellular algae to higher plants), as well as some protists, eubacteria and archaea, while Na^+^-PPases have been identified in some archaea, eubacteria and marine photosynthetic protists. These proteins establish a transmembrane electrochemical gradient, a useful form of biological energy, which might be crucial to overcome stress situations (Serrano et al., 2007). PPi also participates in inositol pyrophosphates (PP-InsPs), a group of signaling molecules found in fungi, plants and animals, involved in the control of Pi homeostasis by interacting with the so-called SPX domains of certain proteins (Shears, 2015; Wild et al., 2016).

Phosphate is stored in different forms depending on organisms and tissues: protists (free-living and parasitic) and fungi store polyP mostly in acidocalcisomes and vacuoles, respectively (Docampo et al., 2005), whereas plants store Pi and inositol phosphates in vacuoles or inclusion bodies of vegetative tissues and seeds, respectively, although phytic acid and other Pi-containing metabolites also seem to be involved (Yang et al., 2017).

Living organisms must finely regulate Pi uptake, incorporation to biomolecules, storage, and mobilization and PPi and polyP are known to be implicated in this regulation, although many aspects remain to be established. Consequently, the metabolism of PPi and polyP in plants and microorganisms has major agricultural and environmental implications, due to the role that these organisms play in the biogeochemical cycle of phosphorus.

The ten articles included in this Research Topic will certainly contribute to the knowledge of the biological importance of PPi and polyP in microorganisms and plants. In a review article, Sanz-Luque et al. summarized the information available on metabolism, storage, and function of polyP in photosynthetic microbes (algae and cyanobacteria) and their potential use in bioremediation. In a similar context, the review article of Baker et al. specifically focused on the molecular and genetic aspects of PolyP production in the context of wastewater remediation by microalgae. Austin and Mayer highlighted the advances in understanding the mechanisms of cellular Pi homeostasis maintained through the INPHORS signaling pathway in yeast. In another review article, Holmes et al. summarized recent structural and functional studies on H^+^- and Na^+^-PPases (mPPases) catalytic and cation pumping mechanisms supporting a complex catalytic cycle involving inter-subunit communication and ion channel motions, which opens new perspectives for their modification as agro-technological and clinical targets. In connection with this topic, the opinion article of Baykov presents a revised interpretation of the energy coupling mechanism involved in oxidative and photo phosphorylation based on recent structural and functional data on mPPases, the simplest and most primitive primary ion pumps known so far.

Two articles reporting experiments carried out with the model organism *Arabidopsis thaliana* illustrate the detrimental effects of alterations in the PPi metabolism in plants. Gunji et al. have shown that vacuolar H^+^-PPase plays a key role in PPi homeostasis and plant morphogenesis, so that an excess of PPi restrains cell morphogenesis and alters organ flatness by collapsing lipid and gluconeogenic metabolisms. The article of Fukuda et al. reported that vacuolar H^+^-PPase and cytosolic soluble PPases act in concert to finely regulate PPi homeostasis, so that lack of these pyrophosphatases causes fatal morphological defects in early stages of plant development. In another article Regmi et al. have proven that overexpression of *Arabidopsis* vacuolar H^+^-PPase (AVP1) in wheat (*Triticum aestivum*) plants improves biomass yield and photosynthate partitioning, a biotechnological strategy that could help to improve crop productivity. In this regard, the article of Pérez-Castiñeira and Serrano demonstrate that mPPases primarily act by hydrolyzing cytosolic PPi when expressed in yeast, and that the *Arabidopsis* vacuolar H^+^-PPase AVP1 is more susceptible to Na^+^ inhibition than the archaeal Na^+^-PPase MVP both *in vivo* and *in vitro*; based on this experimental evidence the use of Na^+^-PPases as biotechnological tools to generate salt-tolerant plants is proposed. Finally, Terashima et al. describe a novel and straightforward method for screening and isolation of polyP accumulating bacteria from complex microbial communities, by using DAPI staining and fluorescence-activated cell sorting.

In conclusion, this collection of themed articles enriches our knowledge on the biological functions of inorganic phosphate polymers, reinforcing the relevance of futures studies aimed to agrotechnological and biomedical applications.

## Author Contributions

All authors listed have made a substantial, direct and intellectual contribution to the work, and approved it for publication.

## Conflict of Interest

The authors declare that the research was conducted in the absence of any commercial or financial relationships that could be construed as a potential conflict of interest.
